# Comparing Acute Effects of a Nano-TiO_2_ Pigment on Cosmopolitan Freshwater Phototrophic Microbes Using High-Throughput Screening

**DOI:** 10.1371/journal.pone.0125613

**Published:** 2015-04-29

**Authors:** Chu Thi Thanh Binh, Christopher G. Peterson, Tiezheng Tong, Kimberly A. Gray, Jean-François Gaillard, John J. Kelly

**Affiliations:** 1 Department of Biology, Loyola University Chicago, Chicago, Illinois, United States of America; 2 Institute of Environmental Sustainability, Loyola University Chicago, Chicago, Illinois, United States of America; 3 Department of Civil and Environmental Engineering, Northwestern University, Evanston, Illinois, United States of America; VIT University, INDIA

## Abstract

Production of titanium-dioxide nanomaterials (nano-TiO_2_) is increasing, leading to potential risks associated with unintended release of these materials into aquatic ecosystems. We investigated the acute effects of nano-TiO_2_ on metabolic activity and viability of algae and cyanobacteria using high-throughput screening. The responses of three diatoms (*Surirella angusta*, *Cocconeis placentula*, *Achnanthidium lanceolatum*), one green alga (*Scenedesmus quadricauda*), and three cyanobacteria (*Microcystis aeruginosa*, *Gloeocapsa *sp., *Synechococcus cedrorum)* to short-term exposure (15 to 60 min) to a common nano-TiO_2_ pigment (PW6; average crystallite size 81.5 nm) with simulated solar illumination were assessed. Five concentrations of nano-TiO_2_ (0.5, 2.5, 5, 10, and 25 mg L^-1^) were tested and a fluorescent reporter (fluorescein diacetate) was used to assess metabolic activity. Algae were sensitive to nano-TiO_2_, with all showing decreased metabolic activity after 30-min exposure to the lowest tested concentration. Microscopic observation of algae revealed increased abundance of dead cells with nano-TiO_2_ exposure. Cyanobacteria were less sensitive to nano-TiO_2_ than algae, with *Gloeocapsa *showing no significant decrease in activity with nano-TiO_2_ exposure and *Synechococcus *showing an increase in activity. These results suggest that nanomaterial contamination has the potential to alter the distribution of phototrophic microbial taxa within freshwater ecosystems. The higher resistance of cyanobacteria could have significant implications as cyanobacteria represent a less nutritious food source for higher trophic levels and some cyanobacteria can produce toxins and contribute to harmful algal blooms.

## Introduction

Nano-TiO_2_ is one of the most widely used engineered nanomaterials with a broad range of commercial and industrial applications [1–3], and its production and use are increasing rapidly [4]. One of the most common applications of nano-TiO_2_ is as a pigment in paints [5], personal-care products (e.g. cosmetics, toothpaste, shampoo, lotions) and food (e.g. milk, candies, sweets, chewing gum, salad dressing) [2,6]. All of these products have the potential to release nano-TiO_2_ to the environment, leading to significant concerns about the possible environmental effects of these materials. Aquatic habitats are especially at risk for nano-TiO_2_ contamination due to inputs from urban and suburban runoff and human wastewater [7–9].

Nano-TiO_2_, particularly anatase, is cytotoxic due mainly to its production of reactive oxygen species (ROS) when illuminated [10], and the toxicity of nano-TiO_2_ has been demonstrated for a variety of aquatic organisms, including fish [11–13], invertebrates [14–16], bacteria [15–18] and algae [19,20]. Algae are critical members of many freshwater ecosystems due to their role as primary producers at the base of the food web. Previous studies have shown that nano-TiO_2_ exposure can inhibit growth and decrease viability for several algal species, including *Scenedesmus* sp. and *Chlorella* sp. [21–23], *Chlamydomonas reinhardtii* [24,25], and *Pseudokirchneriella subcapitata* [26–28]. Most assessments of algal responses to nano-TiO_2_ have exposed algae to nano-TiO_2_ in artificial growth media, but the differences in physicochemical characteristics between growth media and natural surface waters (e.g. organic carbon concentration and ionic strength) could affect nano-TiO_2_ properties such as aggregation [29,30]. Thus, results from studies conducted using artificial growth media might not be representative of microbial responses in natural surface waters. A recent study addressed this issue by using soil extract media as a proxy for natural surface water and demonstrated no significant relationship between growth rate (in 25 day batch cultures) and nano-TiO_2_ concentration for 10 species of freshwater phytoplankton [31]. The use of growth rate as the response variable in determining nano-TiO_2_ effects on algae requires long incubation times (days to weeks) and limits the number of nanomaterials, concentrations and replicates that can be analyzed in parallel. Several recent studies have demonstrated the utility of rapid, high-throughput screening for determining acute bacterial responses to engineered nanomaterials including nano-TiO_2_ [10,17,18,32–35], but this approach has received limited application to algae.

Cyanobacteria can also be significant contributors to primary production in freshwater ecosystems [36], although cyanobacteria are a less nutritious food source than algae for aquatic consumers due to their higher carbon to nitrogen ratio and lower digestibility [37]. Cyanobacteria are also significant because some taxa can produce toxins, and as a result cyanobacterial blooms can be a significant hazard to human and animal health [38]. Few studies have explored the effects of nano-TiO_2_ on cyanobacteria, although one recent study demonstrated that nano-TiO_2_ exposure inhibited the growth rate and nitrogen fixation activity of the cyanobacterium *Anabaena variabilis* [39] and another reported no significant relationship between growth rate and nano-TiO_2_ concentration for *Anabaena* and *Oscillatoria* [31]. Previous studies have demonstrated that cyanobacteria are more resistant to a variety of stressors than algae, including antimicrobial compounds [40,41], but direct comparisons of algal and cyanobacterial responses to nano-TiO_2_ exposure have been limited.

The goal of this study was to investigate the acute effects of a common nano-TiO_2_ pigment on cosmopolitan freshwater phototrophic microbes, including both algae and cyanobacteria, using a high-throughput screening approach with an environmentally relevant medium. Specifically, we assessed the responses of three diatom species (*Surirella angusta*, *Cocconeis placentula*, *Achnanthidium lanceolatum*), one green algal species (*Scenedesmus quadricauda*), and three species of cyanobacteria (*Microcystis aeruginosa*, *Gloeocapsa* sp., *Synechococcus cedrorum*) to short-term exposure to Pigment White 6 (PW6) in Lake Michigan water under simulated solar irradiation. Metabolic activity of the microorganisms was measured with a fluorescence-based esterase activity assay, and microscopic observation was also used to assess cell damage for the algae.

## Materials and Methods

### Water matrix and nano-TiO_2_

Lake Michigan water (LMW) was collected in Evanston, IL and transported to the lab on ice in a cooler. LMW was filtered through 0.22 μm Millipore filters and stored at 4°C in the dark. Characterization of LMW was reported in our previous study [10].

The nanotitania pigment Pigment White 6 (PW6) was purchased from J.T. Baker (Cat. 4162-01). This PW6 was previously characterized as 100% anatase with an average crystallite size of 81.5 nm and an average aggregate size in LMW of 303 nm [10], and its aggregation kinetics in LMW were previously reported [17]. Nano-TiO_2_ stock solutions were prepared by adding 2 g of PW6 to 1 L of Milli-Q water. Stock solutions were sonicated in an ultrasonic bath (Health-Sonics, 110W, 42 kHz) for 30 min and then diluted with Milli-Q water to the desired concentrations for the high-throughput assay. Five dilutions were prepared to give final concentrations in the high-throughput assay of 0.5, 2.5, 5, 10, and 25 mg L^-1^.

### Microbial cultures

Seven phototrophic microbial species commonly found in freshwater ecosystems were obtained from the Culture Collection of Algae at the University of Texas at Austin (Austin, TX, USA): *Achnanthidium lanceolatum* (UTEX LB FD178), *Cocconeis placentula var*. *lineata* (UTEX LB FD44), *Surirella angusta* (UTEX LB FD82), *Scenedesmus quadricauda* (UTEX LB 614), *Synechococcus cedrorum* (UTEX LB 1191), *Microcystis aeruginosa* (UTEX LB 2061), and *Gloeocapsa* sp. (UTEX LB 1938). *A*. *lanceolatum*, *C*. *placentula*, *S*. *angusta*, and *S*. *quadricauda* were cultured in Chu’s #10 medium. *S*. *cedrorum* was cultured in BG-11, and *M*. *aeruginosa* and *Gloeocapsa* were cultured in Bold 3N medium. All cultures were incubated at 20°C for 4 weeks with light intensity of 8.8 W m^-2^ and on a 14:10 hour light: dark photo cycle. Growth of all strains was monitored during the incubation and at week 4 all strains were in exponential growth phase and the cells looked healthy under the microscope. Cells were harvested from each culture by centrifugation and washed twice with filtered LMW. Cells were then resuspended in filtered LMW and adjusted to an optical density at 600 nm (OD_600_) of approximately 0.3 and immediately used for the high-throughput assay. Preliminary experiments indicated that this cell density was required to produce an adequate signal in the HTS assay.

### High-throughput screening assessment of metabolic activity

High-throughput screening (HTS) was used to assess the effect of PW6 on the metabolic activity of the phototrophic microorganisms, with fluorescein diacetate (FDA; Molecular Probes, Life Technologies, Grand Island, NY) used as an indicator of esterase enzyme activity. FDA is colorless, non-fluorescent and membrane permeable, and when FDA enters a cell it is hydrolyzed by nonspecific intracellular esterases resulting in the production of fluorescein, which is fluorescent and not membrane permeable. FDA has been used for decades as an indicator of metabolic activity in algae and has been shown to correlate with photosynthetic activity [42], but it has not been used previously for HTS. In the HTS assay 25 μL of cell suspensions in LMW (prepared as described above) and 25 μL of PW6 solutions (prepared as described above to give final concentration in the assay of 0, 0.5, 2.5, 5, 10, and 25 mg L^-1^) were added to individual wells of 384-well clear-bottom microwell plates using a robotic liquid handler (Biomek FX, Beckman Coulter). Four replicate wells were used for each treatment. Each plate was incubated at room temperature (approximately 22°C) with shaking at 300 rpm on an orbital shaker and illuminated using a xenon arc lamp emitting simulated solar illumination (Model 6271, Newport) at 280–849 nm with an intensity of approximately 200 W m^-2^. The spectral distribution of the lamp was reported in an earlier publication [10]. The measured light intensity and spectrum of the light source were comparable to those of natural solar irradiation [43,44]. Illumination for 60 min increased the temperature within the wells by 3–4°C. To avoid evaporation each plate was covered with an ultraclear film (Axygen, UC-500) that has been shown previously to have a negligible effect on light intensity and spectrum distribution [10]. We also tested in parallel the effect of nano-titania without illumination by covering a section of each microwell plate with aluminum foil. After 15, 30 or 60 min of incubation in the HTS assay plates, 25 μL FDA solution (prepared as instructed by manufacturer) was added to each well by the robotic liquid handler, producing a final concentration of 10 μM in each well, and the microplates were incubated for 1 h in the dark. A microplate reader (Synergy 4, BioTek) was then used to measure fluorescence (excitation 490 nm and emission 520 nm) in each well. Each experiment included a set of control wells containing the full range of nano-titania concentrations but no cells. These abiotic controls demonstrated that the nano-titania concentrations used in our study had no direct effects on the fluorescent signals produced by FDA. Each experiment included an additional set of control wells containing cells but no FDA, and these controls confirmed that the cells alone did not produce a fluorescent signal under the conditions of the FDA assay. In addition, each experiment included a set of no treatment controls, which contained cells but were not incubated in the microwell plates and were not exposed to the simulated solar illumination. These no-treatment controls enabled assessment of metabolic activity immediately prior to the start of the HTS incubations. Finally, for each microbial species a set of standards was prepared by mixing viable and non-viable cells (20 minutes boiled) at several ratios (100% viable, 80% viable: 20% non-viable, 50% viable: 50% non-viable; 20% viable: 80% non-viable; 100% non-viable). Each standard was added to four replicate wells of the tested microplate after completion of the incubation with PW6. FDA solution was added simultaneously to all treatment and standard wells, and the microwell plates were incubated for 1 h in the dark and fluorescence measured with the microplate reader. Calibration curves were constructed for each species based on the fluorescence intensity of the standards, and the percentage of enzyme activity in each test sample was calculated based on the fluorescence intensity and the calibration curve. All seven species used in this study generated calibration curves with correlation coefficients (r^2^ values) greater than 0.98. The inhibition concentration, defined here as the concentration of nano-TiO_2_ that reduced metabolic activity by 25% (IC25), was calculated for each taxa based on the linear interpolation method [45].

### Assessment of algal viability by light microscopy

After completion of the HTS assay algal cells from several of the experimental wells were collected and observed under light microscope. Specifically, solutions from the 0, 0.5 and 25 mg L^-1^ PW6 wells of were collected with pipets and applied to slides. Slides were observed with a 100X oil immersion objective using a BH-2 microscope (Olympus America, Inc., Center Valley, PA, USA). From one starting point each slide was moved in one direction and the first 100 cells were examined and ranked as either healthy, damaged or dead following a previously published method [46,47]. Briefly, cells with intact cell walls and membranes and abundant chloroplasts were ranked as healthy; cells with reduced chloroplasts and/or damaged cell walls and cytoplasm leaking out from the cells were ranked as damaged; and cells with no cytoplasm remaining within the cell wall were ranked as dead. Three replicate slides were prepared and analyzed for each treatment.

### Assessment of cyanobacterial viability by high-throughput screening

The effect of PW6 on the relative abundance of viable cells was assessed for one cyanobacterial species, *Synechococcus cedrorum*, due to its unique response to nano-TiO_2_ exposure in the metabolic activity assay (see Results section below). The relative abundance of viable *S*. *cedrorum* cells was determined using the HTS platform and the Live/Dead BacLight Bacterial viability kit (Molecular Probes) as described previously [17]. The BacLight kit allows determination of the relative abundance of viable bacterial cells within a culture based on the relative signal intensity of two fluorescent dyes, SYTO9, which is membrane permeable, and propidium iodide (PI), which is not membrane permeable and quenches SYTO9. A change in the ratio of the fluorescent signals produced by SYTO9 (green) and PI (red) indicates a change in the relative number of viable bacterial cells, with viable cells defined as those with an intact cell membrane that is impermeable to PI.

*Synechococcus* was cultured and exposed to PW6 in the HTS platform as described above, but after exposure to PW6 for 15, 30 or 60 min 25 μL of BacLight probe solution (prepared according to manufacturer’s instructions) was robotically added to each well. A set of standards with known ratios of viable and non-viable *Synechococcus* cells was also prepared as described above and added to each microwell plate, with each standard well also receiving the BacLight probe solution. Microplates were incubated for 15 min in the dark and the microplate reader was used to measure green fluorescence (excitation 485 nm and emission 530 nm) and red fluorescence (excitation 485 nm and emission 630 nm) in each well. The ratios of green: red fluorescence intensities were calculated for each well and a calibration curve was constructed based on the green: red fluorescence ratios of the standards. The percentage of viable *Synechococcus* cells in each experimental well was then calculated based on the measured green: red fluorescence ratios in the wells and the calibration curve.

### Statistical analysis

Two-way ANOVA was used to test the effects of PW6 treatment and exposure time on esterase activity and cyanobacterial viability determined via HTS. No transformations were performed because the residuals were homoscedastic and normally distributed. In cases of significant interaction effects, one-way ANOVA was used to test the main effects. Percentages of healthy, damaged and dead algal cells obtained from microscopic observations were arc sine square root transformed and then analyzed by two-way ANOVA and one-way ANOVA as above. In cases of significant treatment effects, Tukey’s post hoc tests were used for pairwise comparisons. All statistical analyses were performed with Systat version 13 (Systat Software, Inc.) and in all cases a p value of 0.05 was considered the threshold for statistical significance.

## Results

### Effect of nano-TiO_2_ on algal metabolic activity assessed by HTS

**Fig 1:**
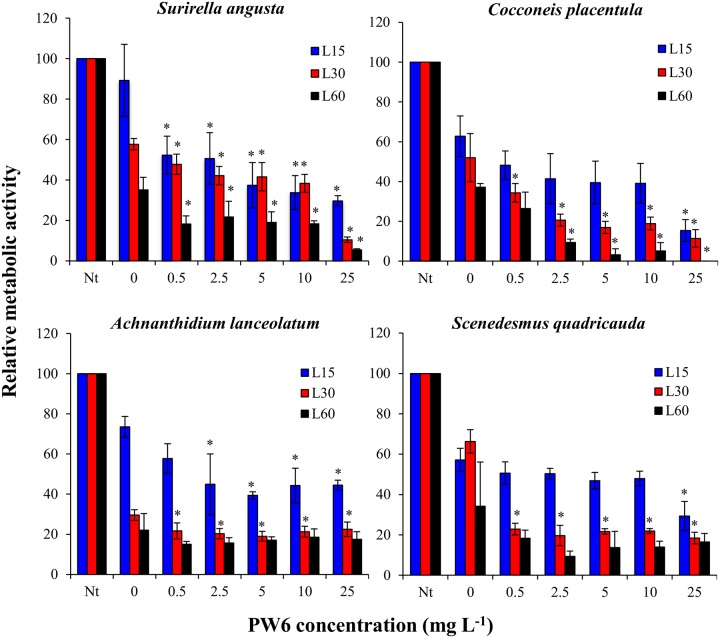
Relative metabolic activity of algae in Lake Michigan water after 15 min (L15), 30 min (L30) and 60 min (L60) exposure under simulated sunlight to nano-TiO_2_ (PW6). Values are reported as mean of 4 replicates ± standard deviation. Nt: no treatment control prior to exposure. Data points marked by asterisks are significantly different (p<0.05) from the 0 mg L^-1^ nano-TiO_2_ control.

Two-way ANOVA indicated significant effects of nano-TiO_2_ treatment and exposure time on metabolic activity for all four algal species tested with the HTS assay (p<0.05), with significant interaction effects for *S*. *angusta*, *A*. *lanceolatum*, and *S*. *quadricauda* (p<0.05), but no significant interaction effect for *C*. *placentula* (p = 0.185). All four algal species were sensitive to simulated solar illumination alone as indicated by the lower activity values in the 0 mg L^-1^ controls as compared to the no treatment (Nt) controls, which were not exposed to light (Fig 1). Therefore, assessments of nano-TiO_2_ effects were based on comparisons between nano-TiO_2_ treatments and the 0 mg L^-1^ controls. All four algal species showed significantly decreased activity after 30 min exposure to the lowest tested nano-TiO_2_ concentration (0.5 mg L^-1^) (p<0.05), and higher nano-TiO_2_ concentrations generally led to further decreases in activity (Fig 1). Exposure time also had a significant effect on metabolic activity for all four algal species (p<0.05) with longer exposure times generally resulting in greater decreases in activity. Illumination was critical to short-term nano-TiO_2_ toxicity, as incubation with nano-TiO_2_ under dark conditions did not result in significant decreases in metabolic activity for any of the algal species relative to the 0 mg L^-1^ controls (S1 Fig). Among the four tested algal species *S*. *angusta* was the most sensitive to nano-TiO_2_ as it was the only species that showed a significant decrease in activity after exposure to the lowest tested nano-TiO_2_ concentration (0.5 mg L^-1^) for only 15 min, and it showed an IC25 for the 60 min incubation of 0.26 mg L^-1^. In contrast *C*. *placentula* and *S*. *quadricauda* were less sensitive to nano-TiO_2_, showing significant decreases in the 15 min exposure only to the highest tested nano-TiO_2_ concentration (25 mg L^-1^). The IC25 values for the 60 min incubations of *C*. *placentula* and *S*. *quadricauda* were 0.43 and 0.27 mg L^-1^, respectively. *A*. *lanceolatum* was intermediate in sensitivity among the four algae, showing a significant decrease in activity in the 15 min exposure at 2.5 mg L^-1^ of nano-TiO_2_ and an IC25 in the 60 min exposure of 0.39 mg L^-1^. Overall the responses of the four algal species to nano-TiO_2_ exposure in the HTS assay followed similar trends of decreased metabolic activity at higher nano-TiO_2_ concentrations.

### Microscopic observation to assess the effects of nano-TiO_2_ on algal cells

**Fig 2:**
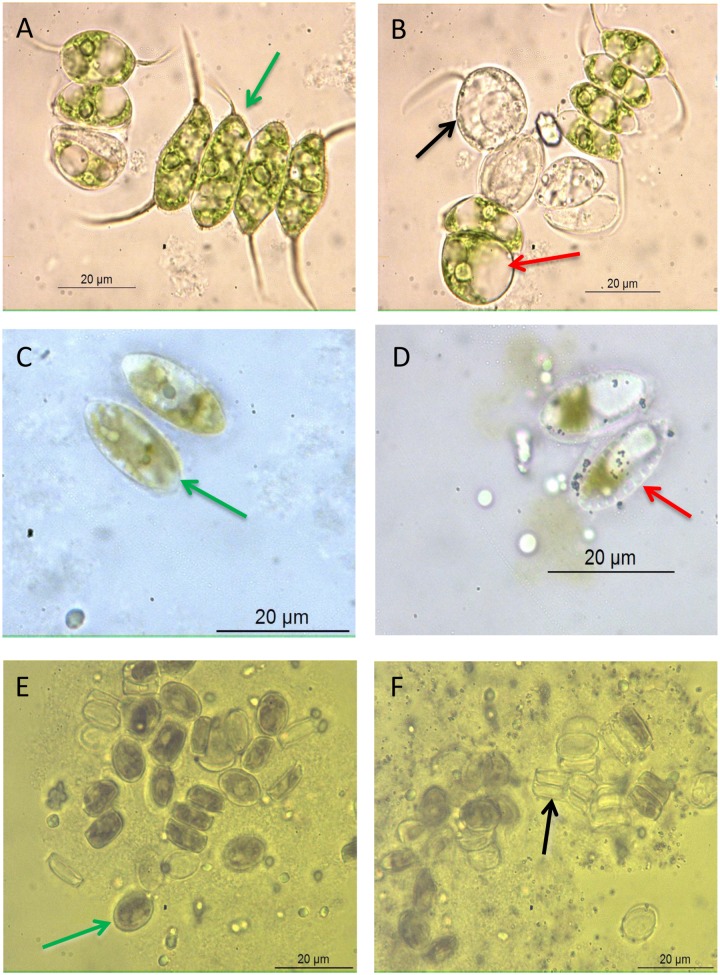
Representative images from microscopic observations of algae after 30 min exposure to simulated sunlight without (A, C, E) and with 25 mg L^-1^ nano-TiO_2_ (B, D, F). Organisms pictured are *Scenedesmus quadricauda* (A & B), *Surirella angusta* (C & D) and *Cocconeis placentula* (E & F). Green arrows indicate examples of healthy cells; red arrows indicate examples of damaged cells; black arrows indicate examples of dead cells.

Cell damage was readily apparent in microscopic observations of *S*. *angusta*, *C*. *placentula* and *S*. *quadricauda* cells that were exposed to nano-TiO_2_ with simulated solar illumination in the HTS assay (Fig 2). For example, healthy *S*. *quadricauda* cells were elliptical and contained abundant green chloroplasts (Fig 2A) whereas exposure to 25 mg L^-1^ nano-TiO_2_ for 30 min resulted in increased size of intracellular vacuoles causing an alteration of cell shape to spherical (Fig 2B). In addition, nano-TiO_2_ treatment resulted in a decrease in the abundance of chloroplasts within *S*. *quadricauda* cells due to leaking of cytoplasm out of the cells, rendering cells transparent (Fig 2B). Similarly, healthy *S*. *angusta* and *C*. *placentula* cells were pigmented (Fig 2C and 2E) and exposure to 25 mg L^-1^ nano-TiO_2_ for 30 min resulted in loss of cytoplasm and chloroplasts presumably due to cell wall damage, leaving empty siliceous frustules (Fig 2D and 2F). Healthy, damaged and dead *A*. *lanceolatum* cells could not be clearly discriminated under the microscope, so microscopic observation data are not reported for this algal species.

**Fig 3:**
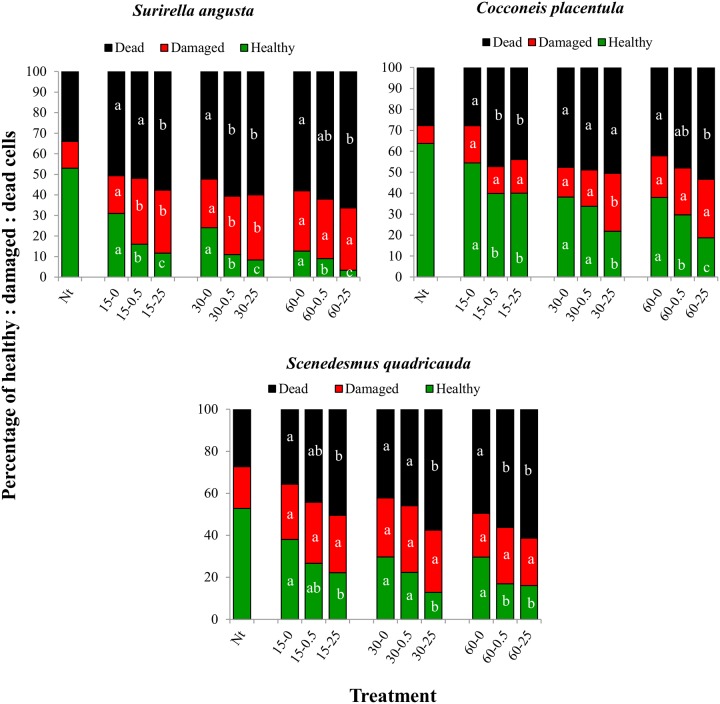
Percentages of healthy, damaged and dead algal cells as identified by microscopic observation after exposure to nano-TiO_2_ under simulated sunlight for 15, 30 and 60 min at three different nano-TiO_2_ concentrations (0, 0.5 and 25 mg L^-1^). The x-axis labels indicate exposure time and nano-TiO_2_ dose, with these numbers separated by a hyphen (e.g. time-dose). All data are mean of 3 replicates. Nt: no treatment control prior to exposure. Data points marked by different letters are significantly different (p<0.05) from the 0 mg L^-1^ nano-TiO_2_ control.

Microscopic observation was used to quantify the abundance of healthy, damaged and dead *S*. *angusta*, *C*. *placentula* and *S*. *quadricauda* cells in selected wells of the HTS metabolic assay (described above). Two-way ANOVA indicated significant effects of nano-TiO_2_ treatment and exposure time on the abundance of healthy, damaged and dead cells for all three algal species (p<0.05), excepting the lack of a significant exposure-time effect on the abundance of damaged cells of *S*. *quadricauda* (p = 0.051). Two-way ANOVA also indicated significant interaction effects between nano-TiO_2_ treatment and exposure time for healthy, damaged and dead cells for *S*. *angusta* and *C*. *placentula* (p<0.05), but no significant interaction effects for *S*. *quadricauda* (p = 0.343 for healthy cells, p = 0.984 for damaged cells and p = 0.774 for dead cells). The trends in responses were similar for all three algal species with nano-TiO_2_ treatment resulting in significant decreases in the numbers of healthy cells, significant increases in dead cells, and almost no significant changes in the abundance of damaged cells (Fig 3). As was observed with the HTS metabolic activity assay, *S*. *angusta* appeared most sensitive among the algal species to nano-TiO_2_ exposure, as this species showed the lowest relative abundance of healthy cells in nano-TiO_2_ treated wells. The relative abundance of healthy *C*. *placentula* and *S*. *quadricauda* also decreased significantly with nano-TiO_2_ exposure, but to a lesser extent than observed with *S*. *angusta*.

### Effect of nano-TiO_2_ on cyanobacterial species assessed by HTS

**Fig 4:**
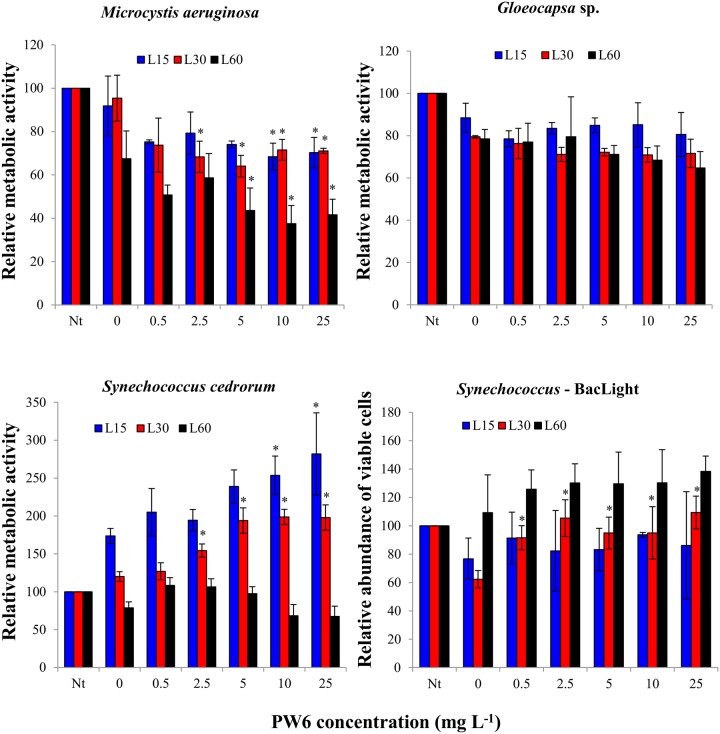
Metabolic activities or relative abundance of viable cells (lower right panel only) of cyanobacteria in Lake Michigan water after 15 min (L15), 30 min (L30) and 60 min (L60) exposure under simulated sunlight to nano-TiO_2_ (PW6). Values are reported as mean of 4 replicates ± standard deviation. Nt: no treatment control prior to exposure. Data points marked by asterisks are significantly different (p<0.05) from the 0 mg L^-1^ nano-TiO_2_ control.

The three tested cyanobacteria, *Microcystis aeruginosa*, *Gloeocapsa* sp. and *Synechococcus cedrorum*, had distinct responses to nano-TiO_2_ exposure in the HTS assay (Fig 4), and none of these cyanobacteria were as sensitive to nano-TiO_2_ as the algal species discussed above (Fig 1). Two-way ANOVA indicated significant effects of nano-TiO_2_ concentration and exposure time on metabolic activity for *M*. *aeruginosa* (p<0.05), but no significant interaction effect (p = 0.271). The metabolic activity of *M*. *aeruginosa* decreased with nano-TiO_2_ treatment, but never fell below 60% of the no-treatment controls for the 30-min exposure and never fell below 40% for the 60 minute exposure, even at a nano-TiO_2_ concentration of 25 mg L^-1^ (Fig 4). The IC25 value for the 60 min exposure of *M*. *aeruginosa* was 2.7 mg L^-1^, which was six to ten-fold higher than the IC25 values for the algal species. Two-way ANOVA indicated no significant effect of nano-TiO_2_ treatment on *Gloeocapsa* (p = 0.729) and no significant interaction between treatment and exposure time (p = 0.989), although there was a significant effect of exposure time (p<0.05), indicating some sensitivity of this species to the simulated solar illumination (Fig 4). Due to the lack of effect of the nano-TiO_2_ treatment on metabolic activity for *Gloeocapsa* an IC25 value could not be calculated for this species. For *S*. *cedrorum*, two-way ANOVA indicated significant effects of nano-TiO_2_ treatment and exposure time on metabolic activity and a significant interaction effect (p<0.05). Remarkably, nano-TiO_2_ exposure resulted in an increase in metabolic activity for *S*. *cedrorum* in the 15 and 30 min exposures and no change in the 60 min exposure (Fig 4). Due to the lack of inhibition of *S*. *cedrorum* metabolic activity by nano-TiO_2_ treatment, an IC25 value could not be calculated for this species. Finally, incubation with nano-TiO_2_ under dark conditions did not result in significant changes in metabolic activity for any of the cyanobacterial species (S2 Fig).

To determine if the observed increase in metabolic activity in *S*. *cedrorum* resulting from exposure to nano-TiO_2_ was based on an increase in cell numbers or simply an increase in the activity of the existing cells, the effect of nano-TiO_2_ on the relative abundance of viable *S*. *cedrorum* cells was determined using HTS with the BacLight assay (Fig 4). Two-way ANOVA indicated no effect of nano-TiO_2_ treatment (p = 0.530), a significant effect of exposure time (p<0.05) and no significant interaction effect (p = 0.553) on the relative abundance of viable *S*. *cedrorum* cells. For the 15 min exposure, one-way ANOVA showed no significant effect of nano-TiO_2_ on the abundance of viable *S*. *cedrorum* cells (p = 0.910), even though the esterase assay had shown a significant increase in metabolic activity (Fig 4), which indicates that the observed increase in metabolic activity was driven by increased activity of the existing cells, rather than by an increase in cell number. For the 30 min exposure, one-way ANOVA indicated that the nano-TiO_2_ treatments showed a significantly higher relative abundance of viable *S*. *cedrorum* cells as compared to the 0 mg L^-1^ control (p<0.05) (Fig 4). However, the abundance of *S*. *cedrorum* cells in the nano-TiO_2_ treatments was not significantly higher than the no-treatment control; rather, the 0 mg L^-1^ control was lower than the no-treatment control. These results indicate that there was not an increase in the number of viable *S*. *cedrorum* cells in the nano-TiO_2_ treatments over the 30 min incubation, so the increase in metabolic activity with nano-TiO_2_ treatment that was observed in the 30 min exposure was driven by increased activity of the existing *S*. *cedrorum* cells, rather than by an increase in cell number. Finally, there was no significant change in the abundance of viable *S*. *cedrorum* cells in the 60 min incubations (p = 0.523), which paralleled the lack of change in metabolic activity of *S*. *cedrorum* cells in the 60 min incubations (Fig 4).

## Discussion

This study aimed to assess the potential ecological effects of nano-TiO_2_ in freshwater ecosystems by measuring the short-term responses of several phototrophic microbial species that are abundant in freshwaters to a common nano-TiO_2_ pigment (PW6). In this study we used a high-throughput screening (HTS) platform, which has been employed by our group and others to assess bacterial sensitivity to engineered nanomaterials [10,17,18,32–35]. We adapted this HTS platform to analyze freshwater phototrophic microbial species, including both algae and cyanobacteria. The HTS method used in this study is based on a fluorescent reporter (fluorescein diacetate; FDA) that measures esterase activity. FDA has been used in previous studies to assess the toxicity of various chemicals to algae and cyanobacteria using flow cytometers [48–50], spectrofluorometers [51,52], and plate readers [51,53]. Our HTS results showed strong correlations between the fluorescence produced by the FDA reporter and the abundance of viable cells for all tested species of algae and cyanobacteria (all correlation coefficients greater than 0.98), indicating that the FDA reporter within the HTS format is an effective indicator of viable phototrophic microorganisms. The HTS assay required cell densities in the range of 1 x 10^6^ to 5 x 10^6^ cells mL^-1^, which is within the range of phytoplankton densities reported for lakes spanning a wide range of physical and chemical conditions [54,55] and is on the low end of values reported for anthropogenically influenced rivers [56,57]. Therefore, the cell concentration used in the HTS assay is environmentally relevant, especially for urban freshwater ecosystems, which are expected to receive nano-TiO_2_ inputs.

One challenge to using HTS to assess the responses of microbes to nano-TiO_2_ exposure is that the toxicity of nano-TiO_2_ is driven primarily by photo-activation of this material that results in production of reactive oxygen species (ROS). However, as demonstrated in this study and our previous work [17], some microbial species are sensitive to light exposure. For example, in the current study all four algal species were sensitive to simulated solar illumination alone, especially for the longer incubation times, as indicated by the lower metabolic activity values in the 0 mg L^-1^ nano-TiO_2_ controls (which were exposed to light) as compared to the no-treatment controls (which were not exposed to light). There are several possible mechanisms that could explain the negative effect of illumination on algal metabolic activity. Exposure to high intensity full spectrum solar radiation (as was used in our HTS assay) can cause photoinhibition and a reduction in photosynthetic activity in algae [58–60]. Photoinhibition is believed to be caused by light-induced damage to the reaction center of Photosystem II [61]. In addition, ultraviolet radiation interacts with dissolved organic matter (DOM) in surface waters producing ROS [62], which are powerful oxidizing agents that can damage a variety of cell components. Thus it is possible that the simulated sunlight used in our HTS assay may have reacted with the DOM in the Lake Michigan water to produce ROS, which in turn damaged the algal cells. Finally, illumination did result in an increase in temperature within the wells (3–4°C), which could have affected the growth of the algae. To account for the negative effect of illumination in our HTS assay, all assessments of nano-TiO_2_ effects in this study and our previous study [17] were based on comparisons between the nano-TiO_2_ treatments and the 0 mg L^-1^ nano-TiO_2_ controls.

The results of the current study demonstrated dramatic differences in the responses of algae and cyanobacteria to acute exposure to nano-TiO_2_ in natural surface water collected from Lake Michigan. All four algal species tested, including three diatoms and one green alga, were highly sensitive to nano-TiO_2_, showing significant decreases in activity after 30 min exposure to the lowest tested nano-TiO_2_ concentration (0.5 mg L^-1^) and IC25 values between 0.26 and 0.43 mg L^-1^ in the 60 min exposures. This suggests a higher sensitivity than we had observed previously for several bacterial species, including *Escherichia coli*, in the same water matrix using the same HTS platform [10,17]. These concentrations of nano-TiO_2_ are higher than has been predicted for aquatic environments [63], but due to the ongoing, exponential increase in nano-TiO_2_ production, nano-TiO_2_ concentrations in the environment are likely to continue to increase [4]. In addition, due to its limited solubility, nano-TiO_2_ will aggregate and settle out of suspension in aquatic habitats [64,65], potentially resulting in long-term accumulation of nano-TiO_2_ in sediments. Disturbance of sediments due to natural events (e.g. storms) or anthropogenic activities could temporarily resuspend nano-TiO_2_ from sediments [64], potentially resulting in high short-term concentrations of nano-TiO_2_ within aquatic habitats.

The decreases in metabolic activity with nano-TiO_2_ exposure observed for the four algal species could be indicative of viable cells decreasing their metabolic activity in response to nano-TiO_2_ exposure, or it could be indicative of cell death due to ROS exposure from nano-TiO_2_. Microscopic observations of *S*. *angusta*, *C*. *placentula* and *S*. *quadricauda* cells collected from the HTS assay demonstrated that nano-TiO_2_ exposure resulted in decreased abundance of viable cells and increased abundance of dead cells for all three species. The decreases in viable cells occurred at the same dosage and incubation times as the reductions in metabolic activity, confirming that cell death contributed to the observed decline in algal metabolic activity with nano-TiO_2_ exposure. Shading due to the absorption of light by TiO_2_ nanoparticles has been suggested as another possible mechanism by which nano-TiO_2_ might decrease the activity of phototrophic microorganisms [31]. However, previous studies have reported that nano-TiO_2_ shading has a negligible effect on algae [19,26].

The three cyanobacterial species tested showed much higher resistance to nano-TiO_2_ exposure than the algae. For example, *Gloeocapsa* showed a remarkable resistance, with no significant change in metabolic activity for any of the tested nano-TiO_2_ concentrations or exposure times, including 25 mg L^-1^ for 60 min. We have observed this level of resistance previously using the HTS format for one bacterial strain, *Arthrobacter sp*. [17], but the resistance of *Gloeocapsa* was much greater than we observed for the algae in this study and for several other bacterial species in prior studies, including *E*. *coli* [10] and *Bacillus subtilis* [17]. *S*. *cedrorum*, another cyanobacteria tested in the current study, was also highly resistant to nano-TiO_2_ exposure, showing no decrease in metabolic activity even when exposed to 25 mg L^-1^ nano-TiO_2_ for 60 min. In fact, for the shorter exposure times (15 and 30 min) *S*. *cedrorum* showed higher metabolic activity with increasing nano-TiO_2_ concentration. This increase in activity with nano-TiO_2_ exposure parallels results of our earlier study in which two bacterial species showed increased abundance of viable cells (i.e. cell growth) with exposure to the same nano-TiO_2_ material (PW6) in stream water using the same HTS format [17]. In that study we showed that exposure of stream water to nano-TiO_2_ with simulated sunlight resulted in photolysis of complex organic compounds in the water, and that stream water pre-treated with nano-TiO_2_ photolysis supported enhanced growth of bacteria, likely due to increased availability of labile carbon and inorganic nutrients [17]. In the current study *S*. *cedrorum* did not experience any significant increase in cell abundance with nano-TiO_2_ treatment, likely due to the relatively short exposure times (15 to 60 min) coupled with the 3 hour generation time of *S*. *cedrorum* [66]. However, the 15 and 30 min nano-TiO_2_ treatments induced significant increases in the metabolic activity of *S*. *cedrorum* cells; we suggest an increase in available nutrients resulting from photolysis of complex organic compounds in the Lake Michigan water by illuminated nano-TiO_2_ as a possible mechanism. This hypothesis is supported by previous studies that have demonstrated the ability of photoactivated TiO_2_ to oxidize organic compounds and release inorganic phosphorous [67] and nitrogen [68]. Cyanobacterial taxa differ in the degree to which growth is stimulated by increased concentrations of inorganic phosphorous and nitrogen [69]. For example, expression of the gene for glutamine synthetase in another freshwater *Synechococcus* species, *S*. *elongates*, has been shown to increase linearly with small increases in the availability of inorganic nitrogen [70]. Together, these observations suggest a possible mechanism for the observed increases in metabolic activity of *S*. *cedrorum* with short-term exposure to nano-TiO_2_, with no such responses noted in the two other cyanbacterial taxa we tested. In contrast to *Gloeocapsa* and *S*. *cedrorum*, metabolic activity in the third cyanobacterial species we tested, *Microcystis aeruginosa*, did decrease significantly with nano-TiO_2_ exposure. This taxon has been shown in previous work by others to be sensitive to TiO_2_ [71]. However, in our study *M*. *aeruginosa* activity decreased only at nano-TiO_2_ concentrations at or above 2.5 mg L^-1^, as compared to 0.5 mg L^-1^ for the algae, and *M*. *aeruginosa* had an IC25 that was six to ten-fold higher than the IC25 values for the algal species. In addition, *M*. *aeruginosa* maintained more than 40% of its activity when exposed to the highest tested nano-TiO_2_ concentration (25 mg L^-1^) for the longest exposure time (60 min); metabolic activities of all algal species we tested were reduced to less than 18% under these conditions. Therefore, while *M*. *aeruginosa* was sensitive to nano-TiO_2_ exposure, it was significantly less sensitive than the four algal species tested.

Another interesting difference between the responses of the algae and cyanobacteria in the HTS assay was that the cyanobacteria were generally less sensitive than the algae to light exposure alone. Cyanobacteria as a group have higher temperature optima than algae [72], so if increased temperature was a key component of the negative effect of illumination in our HTS assay, the lower temperature optima of algae may explain their higher sensitivity to illumination. As mentioned above photoinhibition is another possible mechanism for the negative effect of illumination in our HTS assay, and previous studies have demonstrated photoinhibition in cyanobacteria [73,74]. In the current study *M*. *aeruginosa* and *Gloeocapsa* showed slight reductions in metabolic activity with illumination alone, mainly in the longer exposure times, indicating photoinhibition may have occurred for these species. However, these decreases were not nearly as large as the decreases observed for the algae. Cells of both of these species are enveloped in mucilage—extensively in the case of *Gloeocapsa*. Mucilage has been shown to protect many cyanobacterial taxa, including *Gloeocapsa*, from harmful effects of UV-light [75] and may have been instrumental in the minimal effects of light exposure in these two taxa. Finally, *S*. *cedrorum* showed no significant reduction in metabolic activity with illumination alone, but in fact showed an increase in metabolic activity for the 15 min exposure, possibly due to warming associated with the illumination. *S*. *cedrorum* did show a significant decrease in the relative abundance of viable cells with 30 min exposure to illumination alone, indicating some sensitivity of this species to illumination. Interestingly, a decrease in viable *S*. *cedrorum* cells was not seen in the nano-TiO_2_ treatments. One possible explanation could be that the increase in available nutrients resulting from photolysis of the DOM in the Lake Michigan water by nano-TiO_2_ compensated for the illumination stress. Another possible explanation is that absorption of UV radiation by nano-TiO_2_ provided the *S*. *cedrorum* cells with some protection from damage caused by UV exposure.

The higher nano-TiO_2_ resistance of cyanobacteria as compared to algae may be due to fundamental differences in cell structure, as algae are eukaryotic and cyanobacteria are prokaryotic. For example, cyanobacterial cell walls are composed of peptidoglycan, whereas diatom cell walls are composed of silica and green algal cell walls are composed of cellulose. It is possible that these differences in cell wall structure may contribute to different levels of resistance to ROS. Our microscopic observations of three of the algal species (two diatoms and one green alga) demonstrated that nano-TiO_2_ exposure resulted in rupture of the cell walls and cell membranes and leakage of cytoplasm out of the cell. Cyanobacterial cells, especially *Gloeocapsa* and *S*. *cedrorum* were not negatively impacted by nano-TiO_2_ exposure, suggesting that their cell walls may have been more resistant to ROS. *Gloeocapsa* in particular are known to encase their cells in a thick sheath of mucilage [76] that likely plays a role in protecting the cells from ROS damage. Recent work has also suggested that microcystin, a toxin produced by many cyanobacterial taxa, can help protect cyanobacteria from oxidative stress by binding to proteins involved in photosynthesis and carbon sequestration and protecting them from oxidation [77]. *Microcystis aeruginosa* is a well-known producer of microcystin [78], and a microcystin-producing strain of *Gloeocapsa* was recently isolated from the Salton Sea, in California, USA [79]. Therefore, it is possible that microcystin production could contribute to higher resistance of cyanobacteria to oxidative damage from ROS produced by nano-TiO_2_.

The higher resistance of cyanobacteria to a titanium dioxide nanomaterial (PW6) relative to algae has important ecological implications. Cyanobacteria and algae fill a similar ecological niche, both being aquatic phototrophs, and they compete for resources within aquatic habitats. However, cyanobacteria have a higher carbon to nitrogen ratio and lower digestibility than algae, making cyanobacteria a less nutritious food source for aquatic consumers [37]. Cyanobacteria are also significant because some cyanobacterial taxa can produce toxins and cyanobacterial blooms can be a significant hazard to human and animal health [38]. *Microcystis aeruginosa* is a common component of harmful algal blooms (HABs) and produces microcystin [78], a water soluble toxin that is lethal to many aquatic organisms, including zooplankton and fish [80], and can cause serious damage to the liver of animals, including humans [81]. *Gloeocapsa* has also been identified as a microcystin producer within HABs [79]. Therefore, the higher resistance of cyanobacteria to PW6 that was revealed in our study suggests that exposure to titanium dioxide nanomaterials in aquatic environments has the potential to alter the distribution of phototrophic microbial taxa within freshwater ecosystems in favor of cyanobacteria, which could have significant implications for environmental and human health.

## Supporting Information

S1 Fig Nano-TiO_2_ effects on algae in the dark. Relative metabolic activity of algae in Lake Michigan water after 15 min (red), 30 min (blue) and 60 min (black) exposures to nano-TiO_2_ (PW6) under dark conditions. Values are reported as mean of 4 replicates ± standard deviation.
(PDF)

S2 Fig Nano-TiO_2_ effects on cyanobacteria in the dark. Relative metabolic activity of cyanobacteria in Lake Michigan water after 15 min (red), 30 min (blue) and 60 min (black) exposures to nano-TiO_2_ (PW6) under dark conditions. Values are reported as mean of 4 replicates ± standard deviation.
(PDF)
