# Supplementary material for: Comparing Acute Effects of a Nano-TiO2 Pigment on Cosmopolitan Freshwater Phototrophic Microbes Using High-Throughput Screening
Source: PLoS One. 2015 Apr 29;10(4):e0125613. doi: 10.1371/journal.pone.0125613 (PMC4414569; doi:10.1371/journal.pone.0125613)
Supplement: S2 Fig — Relative metabolic activity of cyanobacteria in Lake Michigan water after 15 min (red), 30 min (blue) and 60 min (black) exposures to nano-TiO2 (PW6) under dark conditions. Values are reported as mean of 4 replicates ± standard deviation. (PDF) [file pone.0125613.s002.pdf]

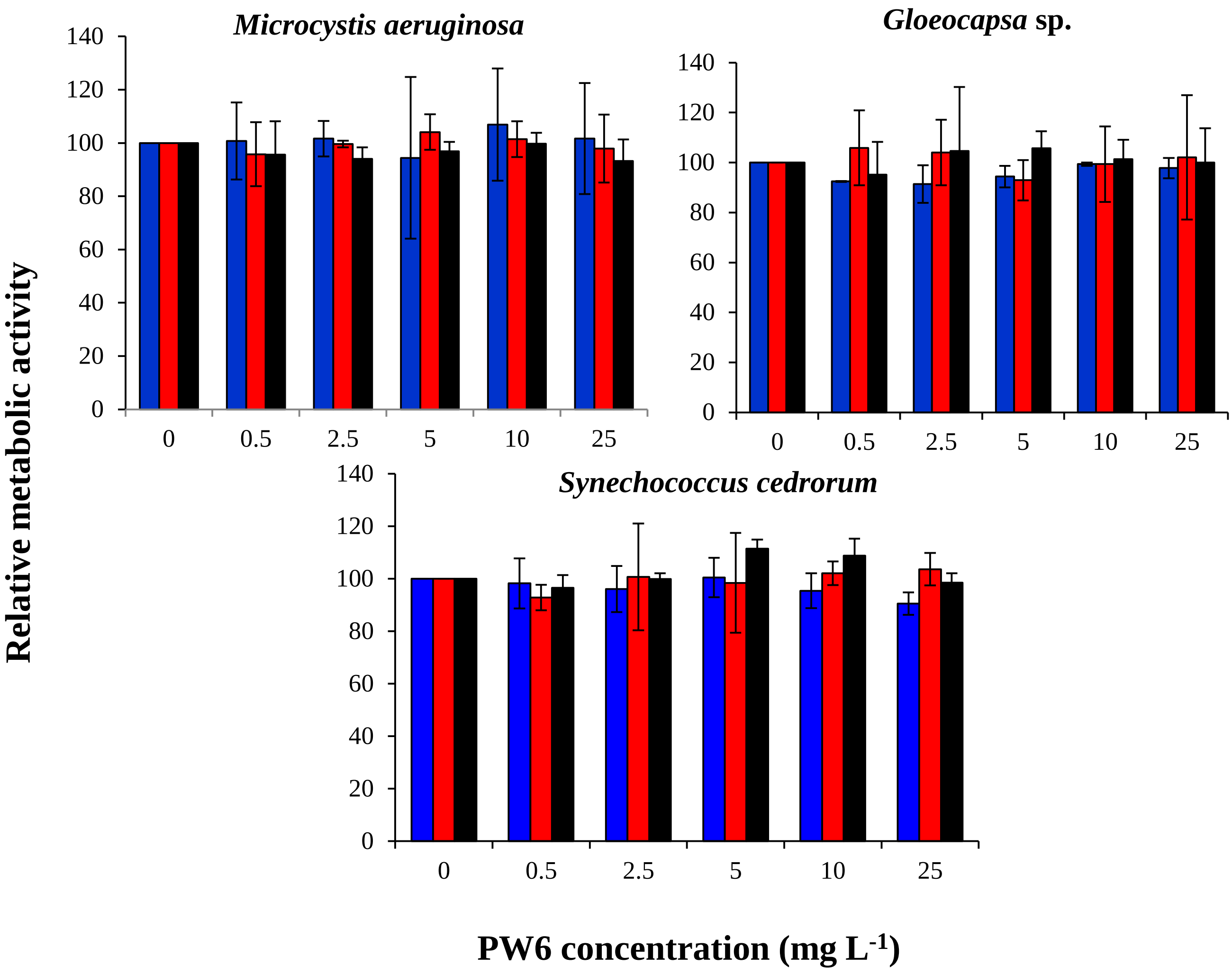

S2 Figure. Nano-TiO_2 effects on cyanobacteria in the dark. Relative metabolic activity of cyanobacteria in Lake Michigan water after 15 min (red), 30 min (blue) and 60 min (black) exposures to nano-TiO_2 (PW6) under dark conditions. Values are reported as mean of 4 replicates ± standard deviation.
